# The evolving role of radiotherapy in oligometastatic pancreatic cancer: from palliation to disease-modifying multidisciplinary integration

**DOI:** 10.3389/fonc.2026.1912332

**Published:** 2026-09-07

**Authors:** Jiarui Zhang, Siming Shi, Jingran Wu, Zhiqiang Tan, Fuliang Liu, Fang Fang, Peng Zhen

**Affiliations:** Department of Radiation Oncology, Chifeng Tumor Hospital, Inner Mongolia Autonomous Region, Chifeng, China

**Keywords:** CtDNA, multidisciplinary integration, oligometastatic pancreatic cancer, patient selection, stereotactic body radiotherapy, TREX1/cGAS-STING

## Abstract

Oligometastatic pancreatic ductal adenocarcinoma (PDAC) may benefit from aggressive multidisciplinary therapy, yet the role of stereotactic body radiotherapy (SBRT) remains controversial and frameworks for patient selection remain incompletely defined. While SBRT has become a standard option in oligometastatic lung and prostate cancer, its application in PDAC faces unique challenges related to tumor biology, anatomic constraints, and a paucity of randomized evidence. This narrative review searched PubMed, Embase, and Cochrane Library (January 2000–May 2026) for English-language studies addressing PDAC, oligometastases, and SBRT. Of 1,247 records screened, 186 studies were included. Six domains were systematically examined: patient selection integrating clinical, molecular, and metabolic imaging biomarkers; pancreas-specific SBRT technical considerations including dose-fractionation and organ-at-risk constraints; systemic therapy integration and sequencing; clinical outcomes and progression patterns; quality-of-life evidence; and ongoing prospective clinical trials. Available evidence from predominantly retrospective series suggests 1-year local control rates of 70–80% for pancreatic primary tumors, 85–95% for lung metastases, and >95% for lymph node metastases, with median overall survival of 12–18 months after SBRT-based consolidative therapy. Emerging biomarkers—including ctDNA dynamics, KRAS mutational subtypes, and transcriptomic classifiers—and advanced technologies such as MR-guided adaptive SBRT and proton therapy show promise for improving the therapeutic ratio. The TREX1/cGAS-STING axis provides a mechanistic rationale for combining SBRT with immune checkpoint inhibitors, though dedicated quality-of-life data remain absent from the literature. Radiotherapy in oligometastatic PDAC has evolved from a purely palliative modality to a disease-modifying component of multidisciplinary care. Prospective validation through pancreas-specific randomized trials incorporating biomarker-driven patient selection and patient-reported outcomes is essential to define the optimal role of SBRT in this setting. Fang Fang and Peng Zhen contributed equally and are co-corresponding authors. Jiarui Zhang and Siming Shi contributed equally to this work.

## 1 Introduction

Pancreatic ductal adenocarcinoma (PDAC) remains one of the most lethal malignancies, with a 5-year overall survival rate of approximately 12% across all stages 9 (1, 2). The majority of patients present with either locally advanced unresectable or metastatic disease, and even among the 15–20% who undergo curative-intent resection, 5-year survival barely exceeds 20–25% (3, 4). For decades, the diagnosis of metastatic PDAC carried an essentially nihilistic outlook, with systemic chemotherapy—first gemcitabine, then FOLFIRINOX, and gemcitabine plus nab-paclitaxel—providing median overall survival improvements from approximately 6 months to 11–12 months (3, 5). Radiotherapy, when used at all, was reserved for palliation of pain, bleeding, or obstructive symptoms.

The conceptual framework for challenging this paradigm emerged from Hellman and Weichselbaum’s 1995 hypothesis of an oligometastatic state—an intermediate phase between localized and polymetastatic disease in which the number and distribution of metastases are sufficiently limited that aggressive local therapy could potentially alter the natural history (4). This hypothesis has since been validated across multiple primary tumor types, most notably by the SABR-COMET phase II randomized trial (with the SABR-COMET-10 randomized phase II trial designed to evaluate the safety and efficacy of SABR for patients with 4–10 metastases (6)), which demonstrated that stereotactic ablative radiotherapy (SABR) to all metastatic sites improved overall survival compared with standard-of-care palliative therapy in patients with controlled primary tumors and 1–5 metastases (median OS 50 months vs 28 months; HR 0.47; p=0.006) (7).

However, PDAC presents unique challenges that distinguish it from the oligometastatic paradigms established in non-small-cell lung cancer, colorectal cancer, and sarcoma. PDAC is characterized by a profoundly immunosuppressive tumor microenvironment rich in regulatory T cells (Tregs), myeloid-derived suppressor cells (MDSCs), and tumor-associated macrophages, with a dense desmoplastic stroma that creates a physical and immunological barrier to both endogenous immunity and therapeutic agents (8). The anatomical proximity of the pancreas to radiosensitive organs at risk (OARs) such as the duodenum, stomach, and small bowel imposes tight dose constraints that limit therapeutic window for SBRT (9). Furthermore, the natural history of PDAC metastases exhibits site-specific heterogeneity—liver-predominant disease portending a distinctly worse prognosis than lung-only or lymph node-only involvement (10).

Recent narrative and systematic reviews have addressed SBRT for oligometastatic disease broadly (11, 13). A comprehensive review of radiation paradigms in pancreatic cancer published in this journal has highlighted the evolving role of SBRT across disease stages, from resectable to metastatic settings (55). However, several critical domains have remained underexplored or absent from the literature: (a) the integration of molecular and liquid-biopsy biomarkers into patient selection, moving beyond simple lesion-count criteria; (b) pancreas-specific biologically effective dose (BED) modeling that acknowledges the distinct α/β ratio and OAR landscape of the upper abdomen; (c) the immunomodulatory effects of SBRT in the PDAC microenvironment and the potential for synergy with immune checkpoint inhibition; and (d) the near-complete absence of quality-of-life and patient-reported outcome data in this population.

This narrative review synthesizes the evidence from 2000 to 2026, with emphasis on developments since 2021, to trace the evolution of radiotherapy in oligometastatic PDAC from symptom palliation toward disease-modifying multidisciplinary integration. We critically examine patient selection, technical optimization, systemic therapy integration, clinical outcomes, and quality-of-life considerations, and propose a framework for future research and clinical practice.

Search Strategy and Selection Criteria: References for this narrative review were identified through searches of PubMed, Embase, and the Cochrane Central Register of Controlled Trials from January 1, 2000 to December 31, 2025. The search strategy combined controlled vocabulary (MeSH, Emtree) and free-text terms for three conceptual domains (1): pancreatic ductal adenocarcinoma (2), oligometastatic disease/neoplasm metastasis, and (3) stereotactic body radiotherapy/stereotactic ablative radiotherapy. Retrieved records (n = 1,247) were screened by title and abstract; full-text articles were assessed for eligibility. Eligible studies included randomized controlled trials, prospective cohort studies, retrospective analyses with ≥20 patients, systematic reviews, meta-analyses, and clinical practice guidelines published in English. Case reports, conference abstracts without full manuscripts, and preclinical studies without clinical correlates were excluded. The final synthesis included 186 studies after full-text eligibility assessment. Given the heterogeneity of study designs and outcomes, a formal meta-analysis was not performed. This review follows a comprehensive (rather than fully systematic) search methodology; formal PRISMA-ScR or PROSPERO registration was deferred given the narrative synthesis approach. The literature search was subsequently updated to include publications through May 2026.

We acknowledge the limitations inherent to a narrative synthesis: (a) selection bias cannot be excluded despite comprehensive search procedures; (b) publication bias favoring positive SBRT outcomes may inflate reported local control rates; (c) several emerging areas—particularly ctDNA-guided therapy and SBRT–immunotherapy combinations—rely on early-phase trials and preclinical data; and (d) Asian-language literature was not comprehensively searched, potentially underrepresenting the substantial East Asian experience with pancreatic SBRT.

## 2 Defining pancreatic oligometastatic disease: clinical patterns and emerging biomarkers

### 2.1 Epidemiological patterns of metastatic PDAC

PDAC most commonly metastasizes to the liver (16) (70–80%), followed by the peritoneum (40–50%), lungs (10–15%), and distant lymph nodes (5–10%) (16, 17). The pattern of metastatic spread carries significant prognostic implications. In a recent analysis of 241 patients with stage IV PDAC and synchronous liver-only metastases, the median overall survival was 7 months overall; patients with resectable liver-only metastases survived significantly longer than those with unresectable disease (15 vs 7 months; p=0.013). These survival benchmarks provide context for evaluating the incremental benefit of local therapies such as SBRT (18).

The distinction between synchronous and metachronous oligometastatic presentation further refines prognostic stratification. Metachronous oligometastases—those developing after a disease-free interval following primary tumor treatment—consistently demonstrate better outcomes than synchronous presentations, in published series (10, 18). This observation likely reflects intrinsic biological differences: metachronous disease that remains oligometastatic after a latency period may represent a less aggressive phenotype with more favorable tumor biology, whereas synchronous oligometastases may indicate inherently aggressive disease that was already disseminating at diagnosis.

### 2.2 Classification frameworks

The European Society for Radiotherapy and Oncology (ESTRO) and European Organisation for Research and Treatment of Cancer (EORTC) consensus classification provides a standardized framework for oligometastatic disease (19). This framework distinguishes five clinical categories:

**Synchronous oligometastases**: Metastases present at initial diagnosis, before primary tumor treatment
**Metachronous oligometastases**: Metastases appearing after definitive primary tumor treatment
**Oligorecurrence**: Metachronous recurrence at a limited number of sites with controlled primary
**Oligoprogression**: Progression at a limited number of sites during systemic therapy, with otherwise controlled disease
**Induced oligometastases**: Polymetastatic disease converted to oligometastatic state after effective systemic therapy—typically a few residual metastases following a marked systemic response—[concept proposed by Palma et al., 2019 (20); formally incorporated into the ESTRO/EORTC classification framework by Guckenberger et al., 2020 (19); reviewed by Hasan et al. (61)].

For pancreatic cancer specifically, the oligoprogression and induced oligometastases categories are particularly relevant in the context of modern systemic therapy, where FOLFIRINOX or gemcitabine/nab-paclitaxel can dramatically shrink tumor burden, occasionally converting what was initially polymetastatic disease into an oligometastatic state amenable to consolidative local therapy (4).

### 2.3 From clinical to molecular patient selection

Historically, patient selection for aggressive local therapy in oligometastatic PDAC has relied on clinical criteria (21): ≤5 metastases, controlled primary tumor, ECOG performance status 0–2, adequate organ function, and a minimum 3–6 month period of disease stability or response on systemic therapy (10, 22). These criteria, derived largely from the oligometastatic literature in NSCLC and colorectal cancer, are a reasonable starting point but lack biological resolution. Not all patients who meet these clinical criteria derive equivalent benefit from SBRT; conversely, some patients who violate these criteria may still benefit.

The last five years have witnessed an accelerating effort to integrate molecular biomarkers into patient selection. Circulating tumor DNA (ctDNA) dynamics have emerged as particularly promising: postoperative ctDNA positivity (23) predicts early recurrence with a lead time of several months over radiographic detection (23), and ctDNA clearance during systemic therapy may identify patients whose disease has been rendered biologically quiescent—a state in which consolidative SBRT is most likely to succeed (24). Prospective validation of ctDNA-guided SBRT selection in PDAC-specific cohorts is essential before clinical implementation (24). KRAS mutation subtype also carries prognostic significance: G12D and G12V mutations exhibit distinct metastatic patterns and organotropism (24), while co-mutations in TP53, SMAD4, and CDKN2A further modulate metastatic behavior—Intact SMAD4 expression is associated with a lower risk of widespread dissemination and a more favorable oligometastatic phenotype (25), and recent data specifically in oligometastatic PDAC patients treated with SBRT suggest that intact SMAD4 expression may predict favorable progression-free survival (26), and TP53 truncating mutations confer a particularly poor prognosis. Emerging data suggest that ctDNA dynamics following radiotherapy for oligometastatic disease may provide prognostic information beyond baseline characteristics, with undetectable post-treatment ctDNA associated with long-term disease control in retrospective series (27, 38). Transcriptomic subtyping (Moffitt basal-like vs classical (28); Collisson quasi-mesenchymal (29)) and 18F-FDG PET/CT metabolic parameters (MTV, TLG (59)) further refine risk stratification, though prospective integration into oligometastatic treatment algorithms remains in its infancy. Transcriptomic analyses have further defined an immunogenic PDAC subtype characterized by upregulated immune networks (60), and mismatch-repair-deficient tumors respond to immune checkpoint inhibition (47), suggesting that only a subset of molecular subtypes may benefit from SBRT–immunotherapy combinations.

It should be emphasized that these molecular biomarkers remain investigational for prospective treatment selection in oligometastatic PDAC. ctDNA dynamics have been evaluated predominantly in retrospective series; no randomized trial has demonstrated that ctDNA-guided SBRT selection improves outcomes over clinical criteria alone. Similarly, KRAS subtyping and transcriptomic classifiers are prognostic rather than predictive in PDAC and have not been shown to predict differential SBRT benefit. Until validated in prospective, histology-specific trials, their role in routine treatment allocation should be considered investigational.

### 2.4 The current selection framework

Integrating these emerging biomarkers into a practical clinical framework, we propose a four-tier approach to patient selection for SBRT in oligometastatic PDAC:

Tier 1 (Clinical-Anatomical): Metastatic pattern (synchronous vs. metachronous), number of lesions, site of involvement (lung-only vs. liver-only), ECOG performance status, and response to induction chemotherapy (CA19–9 kinetics, radiographic stability over a minimum 3–6 month observation window). Tier 2 (Molecular-Biomarker): ctDNA dynamics (clearance during induction therapy), KRAS mutation subtype (G12D vs. G12V), SMAD4/DPC4 expression status, and Moffitt/Collisson transcriptomic classifier (basal-like vs. classical). Tier 3 (Metabolic-Imaging): 18F-FDG PET/CT parameters including metabolic tumor volume (MTV) and total lesion glycolysis (TLG), diffusion-weighted MRI biomarkers where available. Tier 4 (Technical Feasibility): OAR geometry relative to target lesions (duodenum, stomach, small bowel proximity), respiratory motion amplitude on 4D-CT, and availability of advanced delivery platforms (MR-guided adaptive SBRT, proton therapy). Patients meeting favorable criteria across all four tiers are candidates for consolidative SBRT; those failing one or more tiers proceed with systemic therapy and are reassessed at 3-month intervals for possible conversion to eligibility.

## 3 Technical aspects of radiotherapy for pancreatic oligometastases

The application of SBRT to pancreatic oligometastases must contend with the anatomical and biological particularities of the upper abdomen. Liver, lung, and nodal metastases each present distinct dosimetric considerations, and the primary pancreas tumor itself—when targeted as part of an oligometastatic treatment plan—carries unique challenges related to target delineation, motion management, and normal tissue sparing that differ substantially from the oligometastatic paradigms established in thoracic and musculoskeletal oncology.

### 3.1 Target volume definition and simulation

Accurate target delineation for pancreatic oligometastases begins with high-quality simulation imaging. For liver metastases, multiphasic contrast-enhanced CT (triple-phase or quadruple-phase) co-registered with contrast-enhanced MRI—preferably with hepatobiliary phase imaging using gadoxetic acid—provides superior lesion visualization and is recommended whenever available (30). For pancreatic primary tumors, dual-phase pancreatic protocol CT with thin-slice (1–2 mm) acquisition, often supplemented by MRI/MRCP and endoscopic ultrasound, is essential for accurate gross tumor volume (GTV) delineation.

Motion management represents a critical technical consideration, particularly for liver and pancreas targets that are subject to respiratory and gastric motion. Four-dimensional CT (4D-CT) with respiratory phase binning is the standard approach for characterizing tumor motion amplitude and generating an internal target volume (ITV) (33). For pancreatic and liver metastases with excursion >5 mm, active motion management strategies are warranted, including:

**Abdominal compression** to limit diaphragm excursion (typically reduces motion to 3–7 mm)
**Gated delivery** using respiratory surrogates (RPM, real-time positioning management) or fiducial-based tracking (typically reduces treatment volumes by 30–50%)
**Breath-hold techniques** (deep inspiration breath-hold, active breathing control) for highly reproducible target positioning

Fiducial markers placed under endoscopic ultrasound (for pancreatic primary) or CT/ultrasound guidance (for liver metastases) substantially improve daily target localization. A placement of fiducial markers or surgical clips is recommended to improve daily target localization for pancreatic SBRT, with electromagnetic transponder tracking considered an emerging alternative (34).

### 3.2 Dose-fractionation and biologically effective dose optimization

The optimal dose-fractionation regimen for SBRT in pancreatic oligometastases remains an area of active investigation and debate. Available regimens fall broadly into three categories based on the metastatic site and technical capability of the treatment platform. **Table 1** summarizes the most commonly reported regimens.

**Table 1:** Commonly reported SBRT dose-fractionation regimens for oligometastatic PDAC by metastatic site.

Metastatic site	Typical regimens	BED10 (Gy)	Key constraints	Reported 1-year LC
Liver	30–60 Gy/3–5 fx	60-132	>=700 mL <15 Gy; Bowel Dmax <30 Gy/3 fx	70-85%
Lung	48–60 Gy/3–5 fx	100-132	Chest wall Dmax; Proximal bronchus <48 Gy/5 fx	85-95%
Lymph node	30–45 Gy/3–5 fx	60-86	Bowel Dmax; Spinal cord Dmax <18 Gy/3 fx	>95%
Pancreatic primary	33–40 Gy/5 fx	55-72	Duodenum Dmax <35 Gy; Stomach Dmax <35 Gy	70-80%

The linear-quadratic model, though derived from fractionated radiotherapy, provides a useful framework for comparing SBRT regimens when applied with appropriate caveats. Available data suggest an α/β ratio of approximately 8–10 Gy for PDAC, consistent with other adenocarcinomas (35, 36). However, two critical pancreas-specific considerations complicate direct application of BED models derived from lung or liver SBRT:

First, the **proximity to serial OARs with low α/β** (duodenum ~3 Gy, stomach ~3 Gy, spinal cord ~2 Gy) creates a narrow therapeutic window. The duodenum is of particular concern, as late toxicity from duodenal ulceration or bleeding occurs at BED_3_ values exceeding approximately 80 Gy (36). For a 5-fraction SBRT regimen delivering 33–40 Gy to the pancreatic primary, the duodenal Dmax of <35 Gy (conventionally expressed as Dmax or D0.5cc) corresponds to a BED_3_ of approximately 105–117 Gy, approaching the tolerance threshold (37).

Second, **tumor heterogeneity within PDAC**—specifically the variable stromal content and hypoxic fraction—may modulate the effective α/β ratio and thus the BED required for tumor sterilization. Preclinical data suggest that dense desmoplastic stroma increases the effective α/β ratio, potentially reducing the biological effect of high-dose-per-fraction SBRT (39). This observation provides a mechanistic rationale for the ongoing investigation of higher-dose regimens (e.g., 50 Gy/5 fractions with MR-guided adaptation to achieve simultaneous OAR sparing) (40).

### 3.3 Organ-at-risk constraints

The radiation tolerance of upper abdominal organs has been refined over two decades of SBRT experience. Contemporary constraints for pancreatic SBRT, largely derived from large multi-institutional series and meta-analyses, include:

**Duodenum**: Dmax <35 Gy (5 fractions), D1cc <33 Gy; for 3-fraction liver SBRT with lesions near duodenum: Dmax <30 Gy
**Stomach**: Dmax <35 Gy (5 fractions); D1cc <33 Gy
**Small bowel** (non-duodenal): Dmax <33 Gy (5 fractions); loop-specific constraints remain debated
**Liver** (for hepatic metastases): ≥700 mL of healthy liver (liver minus GTV) receiving <15 Gy; ≥40% receiving <10 Gy
**Kidneys** (bilateral): Mean dose <10 Gy for each kidney; total V12 <20%
**Spinal cord**: Dmax <18 Gy (3 fractions); Dmax <22 Gy (5 fractions)
**Chest wall** (for lung/rib metastases): Dmax <50 Gy (5 fractions); V30 <30 cc for rib fracture risk

These constraints are derived predominantly from retrospective series and must be interpreted cautiously. The introduction of MR-guided adaptive SBRT has enabled tighter planning margins (3–5 mm vs 5–10 mm with conventional CT-guided SBRT), with evidence from comparative series suggesting a substantial reduction in Grade 2+ GI toxicity (34, 41).

### 3.4 Advanced delivery technologies

Proton SBRT may offer dosimetric advantages for hepatic metastases, though clinical outcome data in the oligometastatic setting remain limited (42).

### 3.5 Single-fraction vs multi-fraction debate

In the broader oligometastatic SBRT literature, single-fraction regimens (e.g., 18–24 Gy for lung, 18–25 Gy for liver) are common and offer logistical advantages. For pancreatic oligometastases, however, multi-fraction regimens (3–5 fractions) are strongly preferred for several reasons. First, the α/β ratio for PDAC (≈10 Gy) is relatively high among solid tumors, meaning that the fractionation sensitivity of tumor cells is low: there is limited biological penalty for fractionating the tumor dose. Conversely, the serial organs at risk (duodenum, stomach, small bowel) have low α/β ratios (≈3 Gy), making them highly sensitive to dose per fraction. Fractionation therefore exploits the differential α/β between tumor and late-responding normal tissues—sparing OARs while maintaining tumor BED through modest increases in total dose. Second, multi-fraction schedules allow for biological reoxygenation and redistribution between fractions, which may be particularly relevant for PDAC’s characteristically hypoxic microenvironment (39). Third, multi-fraction regimens are more compatible with adaptive workflows, where daily re-planning can correct for inter-fraction anatomical variation—a critical consideration given the proximity of the duodenum and stomach. Fourth, the TREX1/cGAS-STING axis introduces an immunological dimension: single-fraction doses exceeding approximately 12–18 Gy upregulate the DNA exonuclease Trex1, which degrades cytosolic DNA and attenuates STING-dependent type I interferon signaling, paradoxically reducing the immunogenicity of ultra-high single-dose SBRT (12, 14, 15). Moderate hypofractionation (e.g., 6–8 Gy × 3–5 fractions) may preserve STING pathway activation and may enhance the abscopal potential. Recent evidence further suggests that necroptotic signaling through ZBP1-MLKL can potentiate radiation-induced STING pathway activation, providing an additional mechanistic rationale for immunologically optimized fractionation, though this evidence derives from murine models and has not been validated in human PDAC (43). The field has therefore converged on 5-fraction SBRT as the most pragmatic standard for pancreatic primary and liver metastases, with 3-fraction regimens reserved for lung and nodal targets where the OAR geometry is more favorable.

## 4 Integration with systemic therapy: the multidisciplinary core

The central thesis of this review—that radiotherapy’s role in oligometastatic PDAC has evolved from palliation to disease-modifying multidisciplinary integration—is most powerfully illustrated by the complex interplay between SBRT and contemporary systemic therapy. This section examines the three critical dimensions of this integration: chemotherapy backbone, immunotherapy synergy, and targeted therapy opportunities.

### 4.1 Chemotherapy backbone: FOLFIRINOX vs gemcitabine-based regimens

Two systemic regimens form therapeutic backbone for metastatic PDAC. FOLFIRINOX (oxaliplatin, irinotecan, fluorouracil (3), leucovorin) achieved a median OS of 11.1 months compared with 6.8 months for gemcitabine in the landmark phase III trial (5), while gemcitabine plus nab-paclitaxel demonstrated a median OS of 8.5 months vs 6.7 months (3). Although no randomized head-to-head comparison exists, these regimens are associated with different toxicity profiles and, importantly, different radiosensitizing properties.

FOLFIRINOX components have not been demonstrated to possess significant direct radiosensitizing properties comparable to those of gemcitabine (44). The therapeutic sequence that has emerged in practice is induction FOLFIRINOX (4–6 cycles), followed by restaging, then consolidative SBRT to oligometastatic sites in patients with controlled or responding disease. This “chemotherapy-first” paradigm, analogous to the approach validated in locally advanced PDAC by the LAP 07 trial (31), allows the biology of the disease to declare itself over 3–6 months of systemic therapy. Patients who progress polymetastatically during induction chemotherapy are spared the toxicity of SBRT, while those who achieve sustained oligometastatic control become candidates for consolidative local therapy.

Gemcitabine, by contrast, is a potent radiosensitizer through its action as a deoxycytidine analog that depletes deoxyribonucleotide pools and enhances radiation-induced DNA damage. The radiosensitizing effect of gemcitabine peaks during S-phase (44), lasts 48–72 hours after administration, and is schedule-dependent—with weekly low-dose gemcitabine (200–400 mg/m²) producing supra-additive effects without prohibitive toxicity (44). This property has been exploited in multiple phase I/II trials of gemcitabine-based chemoradiation for locally advanced PDAC, with acceptable toxicity and promising local control. In the oligometastatic setting, the radiosensitizing potential of concurrent low-dose gemcitabine with SBRT remains largely unexplored but represents a rational avenue for future investigation, particularly for patients whose systemic therapy backbone is gemcitabine/nab-paclitaxel rather than FOLFIRINOX.

### 4.2 Sequencing strategies

The optimal sequencing of SBRT relative to systemic therapy depends on the clinical context. For synchronous oligometastases, a chemotherapy-first approach—typically 4–6 cycles of FOLFIRINOX or gemcitabine/nab-paclitaxel followed by restaging—is preferred. This sequence, pioneered in pancreas SBRT by Mahadevan et al. (45) and analogous to the LAP07 paradigm (31), exploits a 3–6 month observation window during which patients destined for early polymetastatic progression declare themselves, thereby avoiding futile local therapy. For oligoprogression during maintenance therapy, SBRT to the progressive site(s) while continuing the current systemic regimen is a pragmatic strategy, extrapolated from NSCLC experience (32, 46). For induced oligometastases—where an exceptional systemic response converts polymetastatic to oligometastatic disease—consolidative SBRT to residual sites is a rational but unproven approach that warrants prospective evaluation. Across all scenarios, the overarching principle is that SBRT complements, rather than replaces, effective systemic therapy.

### 4.3 Immunotherapy synergy: a frontier for pancreatic oligometastases

The promise of immunotherapy has transformed oncology, yet PDAC remains a conspicuous exception (8). Single-agent PD-1/PD-L1 checkpoint inhibitors have negligible activity in microsatellite-stable (MSS) PDAC, which constitutes >95% of cases (47). The challenge lies in the PDAC tumor microenvironment (TME): a dense desmoplastic stroma characterized by abundant cancer-associated fibroblasts (CAFs), Tregs, MDSCs, and tumor-associated macrophages that together create a formidable barrier to T-cell infiltration and effector function (39).

However, the immunogenicity of radiation is counterbalanced by immunosuppressive feedback mechanisms. The DNA exonuclease Trex1—induced by radiation doses exceeding approximately 12–18 Gy per fraction—degrades cytosolic DNA and attenuates the cGAS-STING pathway, paradoxically reducing the immunogenicity of higher-dose-per-fraction SBRT (14). This finding has important implications for fractionation selection when SBRT is combined with immunotherapy in PDAC, suggesting that moderate hypofractionation (e.g., 8 Gy × 3 fractions or 6 Gy × 5 fractions) may be immunologically superior to single high-dose fractions (e.g., 20–24 Gy × 1) (14).

Several ongoing clinical trials are building on this foundation. NCT03104439 is evaluating SBRT (6.6–8 Gy × 3–5 fractions) combined with pembrolizumab in oligometastatic PDAC, with the hypothesis that the addition of SBRT to PD-1 blockade will enhance immunologic activation beyond that expected from PD-1 blockade alone. NCT02866383 (CheckPAC) is investigating the combination of SBRT with nivolumab with or without ipilimumab in the second-line or later metastatic setting. However, it must be emphasized that clinical evidence for SBRT-immunotherapy synergy in PDAC remains absent. The phase II trial by Parikh et al. (Nat Cancer 2021) (48) evaluating radiotherapy plus immunotherapy in MSS colorectal and pancreatic adenocarcinoma reported no objective responses in the PDAC cohort, underscoring the formidable barriers posed by the immunosuppressive PDAC microenvironment. Strategies to overcome these barriers—including TGF-beta inhibition, CXCR4 antagonism, and CAR-T cell therapy—are under preclinical and early clinical investigation but have not yet demonstrated meaningful activity in combination with SBRT in PDAC. Results from these trials will be critical in determining whether the immunomodulatory effects of SBRT can be harnessed for clinically meaningful benefit in PDAC.

### 4.4 Targeted therapy integration

The POLO trial established maintenance olaparib as a standard of care for patients with germline BRCA1/2-mutated metastatic PDAC (49) who have not progressed after 4+ months of first-line platinum-based chemotherapy, demonstrating a progression-free survival benefit of 7.4 months vs 3.8 months (HR 0.53) (49). The combination of PARP inhibition with SBRT is biologically rational: PARP inhibitors impair DNA damage repair through the base-excision repair pathway, and the radiosensitizing effect of PARP inhibitors effect is well documented in preclinical models (50). In the oligometastatic setting, SBRT to oligoprogressive sites during olaparib maintenance therapy could potentially extend the duration of benefit from PARP inhibition, though this strategy must be pursued cautiously due to the risk of enhanced normal tissue toxicity. Clinical trials formally evaluating the PARPi + SBRT combination in PDAC are lacking and represent a high-priority area for investigation.

### 4.5 The multidisciplinary framework

The integration of SBRT into the management of oligometastatic PDAC is fundamentally a multidisciplinary endeavor. We propose a practical decision framework:

This framework integrates clinical stratification (synchronous versus metachronous, metastatic site), molecular biomarker profiles, metabolic imaging characteristics, and technical feasibility to guide SBRT decision-making. Evidence levels reflect the current literature: Level II from retrospective cohort studies; Level III from expert consensus or extrapolation from NSCLC; Level IV from preclinical rationale. This framework is hypothesis-generating and requires prospective validation.

## 5 Clinical outcomes, toxicity, and quality of life

### 5.1 Local control by metastatic site

The EXTEND trial represents the first randomized evidence specifically in oligometastatic PDAC, demonstrating that the addition of comprehensive metastasis-directed therapy (MDT) to systemic chemotherapy significantly improved progression-free survival compared with chemotherapy alone (51). However, it should be noted that overall survival benefit has not yet been demonstrated, and the trial enrolled a highly selected cohort of patients with indolent disease biology. These results, while encouraging, highlight the gap between PFS improvements and definitive OS gains in this population. A recent SEER-validated cohort analysis published in this journal further demonstrated that combined-modality therapy including radiotherapy significantly improved overall survival compared with chemotherapy alone in unresectable and metastatic pancreatic cancer (median OS 22.2 vs 11.8 months after propensity score matching; HR 0.49, P = 0.003), reinforcing the rationale for integrating local radiotherapy into systemic treatment paradigms (56).

The primary objective of SBRT in oligometastatic PDAC is durable local control of irradiated lesions. Available data, derived predominantly from retrospective institutional series and pooled analyses, indicate site-dependent differences in local control rates. A recent institutional series of 62 patients with oligometastatic pancreatobiliary cancer reported 12-month local control of 78.5% with no grade ≥3 toxicity after SBRT (52).

### 5.2 Overall survival

The interpretation of overall survival (OS) data for SBRT in oligometastatic PDAC is complicated by inherent selection bias in retrospective series, as patients selected for SBRT are systematically healthier than those treated with systemic therapy alone. With this caveat, available data suggest median OS of 12–18 months after SBRT for pancreatic oligometastases (51), compared with historical controls of 9–12 months for metastatic PDAC treated with modern chemotherapy alone (3, 5). This represents a potential improvement of 3–8 months, consistent with the effect size observed in the SABR-COMET trial across all histologies (7).

Factors consistently associated with favorable OS after SBRT include:

- Metachronous (vs synchronous) presentation
- Lung-only (vs liver-only) metastatic pattern
- Single (vs multiple) metastatic lesion
- ECOG 0 (vs 1–2) performance status
- CA19–9 response (>50% decrease) after induction chemotherapy
- ctDNA clearance during induction therapy (emerging data)

### 5.3 Progression patterns

It is noteworthy that radical surgical resection of oligometastatic PDAC (53)—including both primary tumor and synchronous hepatic or pulmonary metastases—has been explored in highly selected cohorts, with retrospective series reporting 5-year survival rates of 8–15% in patients undergoing complete macroscopic resection (53, 54). These surgical data provide a benchmark against which SBRT outcomes should be contextualized, though direct comparisons are confounded by selection bias favoring surgical candidates with more favorable performance status and disease biology.

Understanding where and when disease progresses after SBRT is critical for optimizing treatment strategies. Available data suggest that most first progression events occur at distant (non-irradiated) sites, with a minority representing in-field or marginal recurrences. The predominance of distant progression underscores a fundamental limitation of SBRT: local therapy, no matter how effective, cannot control occult micrometastatic disease that was already present at the time of treatment. This observation reinforces the essential role of effective systemic therapy as the backbone of any oligometastatic treatment strategy, with SBRT serving as a consolidative adjunct rather than a substitute.

### 5.4 Toxicity profile

The toxicity of SBRT for pancreatic oligometastases depends critically on the treated site, the dose-fractionation regimen, and the proximity to OARs.

For pancreatic primary SBRT, the proximity of the duodenum represents the dose-limiting constraint. It should be noted that the toxicity data cited below derive predominantly from locally advanced PDAC SBRT series; while the dosimetric principles are transferable, toxicity rates specific to the oligometastatic setting are less well characterized. Duodenal toxicity (36)—including ulceration, bleeding, and perforation—occurs at rates of 5–15% in contemporary series employing 5-fraction SBRT regimens (33–40 Gy), with grade 3+ events reported in 3–8% of patients. Delineation of the duodenum on daily MR-guided imaging and the use of adaptive re-planning have been associated with a substantial relative reduction in grade 2+ gastrointestinal toxicity (34, 41). For liver SBRT, radiation-induced liver disease (RILD) is rare with modern techniques (≤1% in series using strict liver sparing constraints), though transient grade 1–2 transaminitis occurs in 15–25% of patients. For lung SBRT, symptomatic pneumonitis (grade 2+) occurs in <5% for small-volume disease. In a multi-institutional phase II trial of gemcitabine plus SBRT (33 Gy/5 fractions), late grade ≥2 gastrointestinal toxicity was 11% with acceptable treatment tolerability with symptom improvement (57).

### 5.5 Quality of life: the neglected endpoint

The EORTC QLQ-C30, validated in international clinical trials (58), remains the most widely used instrument for health-related quality-of-life assessment in pancreatic cancer studies. However, dedicated QoL data following SBRT specifically in the oligometastatic PDAC population remain conspicuously absent from the literature, representing a critical gap that future prospective trials must address through systematic incorporation of patient-reported outcome measures.

## 6 Ongoing clinical trials and future directions

### 6.1 Key ongoing trials

The therapeutic landscape for oligometastatic PDAC is evolving rapidly, with several ongoing trials addressing critical knowledge gaps. The SMART master protocol (NCT04115254) is evaluating MR-guided online-adaptive SBRT across pancreatic, lung, renal, and liver metastases, and the OLIGOMETS trial (NCT07282912) is testing the addition of sequential local consolidative therapy to standard systemic therapy in foregut adenocarcinomas. Early-phase studies are also combining SBRT with pembrolizumab and hypofractionated radiotherapy (RADVAX, NCT02303990) or with dual immune checkpoint blockade (NCT02311361). **Table 2** summarizes the most impactful ongoing and completed studies.

**Table 2:** Key clinical trials of SBRT in oligometastatic PDAC and related immunotherapy combinations.

NCT number	Trial name/acronym	Phase	Population	Intervention	Status
NCT03599765	EXTEND	II (randomized)	Oligometastatic solid tumors, incl. PDAC basket (n=40)	Systemic therapy ± local consolidative therapy (SBRT)	Completed (results published; Ludmir 2024)
NCT03104439	—	II	MSS CRC and PDAC (n=25 PDAC)	Pembrolizumab + RT (24 Gy/3 fx)	Completed (results published; Parikh 2021)
NCT02311361	—	I	Unresectable pancreatic cancer (n=59)	Durvalumab ± tremelimumab + SBRT (8 Gy×1 or 5 Gy×5)	Completed; published (Xie 2020); results on CT.gov
NCT04115254	SMART	I/II (master protocol)	Pancreas, lung, renal, liver metastases, oligometastatic nodal disease, etc.	MR-guided adaptive SBRT (MR-Linac)	Recruiting; est. completion 2028
NCT07282912	OLIGOMETS	II (randomized)	Foregut adenocarcinoma (esophageal, gastric, pancreatic, biliary) with undetectable ctDNA after induction chemo	Standard therapy ± sequential cytoreductive intervention (surgery/SBRT/ablation)	Recruiting; started Mar 2026
NCT02866383	CheckPAC	II (randomized)	Metastatic pancreatic or biliary tract cancer	Nivolumab ± ipilimumab + high-dose RT	Completed
NCT02303990	RADVAX	I	Advanced/metastatic solid tumors incl. pancreatic, breast, NSCLC, melanoma	Pembrolizumab + hypofractionated RT	Completed; results on CT.gov
NCT05411094	—	I	Locally advanced unresectable pancreatic cancer	Olaparib + durvalumab + RT	Suspended

### 6.2 Priority research directions

First, the field urgently requires a pancreas-specific phase II/III randomized trial of SBRT for oligometastatic PDAC. The recently reported EXTEND trial (NCT03599765), which randomized patients with oligometastatic PDAC to chemotherapy alone versus chemotherapy plus comprehensive metastasis-directed therapy, provides the first randomized evidence supporting the oligometastatic paradigm specifically in PDAC, demonstrating a statistically significant improvement in progression-free survival with the addition of MDT (51). Larger-scale validation is now essential.

Second, ctDNA-guided adaptive strategies represent the next frontier in precision patient selection. Trials incorporating ctDNA clearance as an eligibility criterion for consolidative SBRT will determine whether molecular response can outperform clinical and radiographic criteria in identifying the subset of patients most likely to benefit.

Third, the optimal integration of SBRT with immunotherapy in PDAC requires systematic exploration. While preclinical data support synergy (14, 15), clinical translation has been hampered by the immunosuppressive PDAC microenvironment. Rational combination strategies—including the use of moderate hypofractionation to preserve STING pathway activation (14), the addition of TGF-β or CSF1R inhibitors to reprogram the stroma, and strategies to overcome the immunosuppressive PDAC microenvironment (e.g., TGF-β or CSF1R inhibition, stromal reprogramming)—merit dedicated investigation.

Fourth, health-related quality of life and patient-reported outcomes must be incorporated as mandatory endpoints in all future prospective trials. The current evidence gap undermines informed decision-making in a disease where median survival remains limited.

Fifth, cost-effectiveness analyses of SBRT in oligometastatic PDAC are entirely absent and are urgently needed to support health technology assessment and reimbursement decisions.

### 6.3 Uncertainties and knowledge gaps

Despite the encouraging trajectory of the evidence reviewed herein, several fundamental uncertainties warrant explicit acknowledgment. First, the oligometastatic paradigm itself has been predominantly validated in non-PDAC histologies; the randomized evidence specific to PDAC remains limited to fewer than 100 patients across the EXTEND trial and the PDAC subgroup of SABR-COMET. The possibility that the apparent survival benefit of SBRT in retrospective series is substantially attributable to selection bias—specifically immortal time bias and the preferential selection of patients with indolent tumor biology—cannot be excluded without adequately powered, histology-specific randomized trials.

Second, the incremental benefit of SBRT must be contextualized against contemporary systemic therapy benchmarks. The historical chemotherapy comparators cited in this review (median OS 9–12 months from the FOLFIRINOX and gemcitabine/nab-paclitaxel era) may underestimate current outcomes achievable with modern triplet regimens such as NALIRIFOX. Without a contemporary systemic therapy control arm, the true incremental benefit of SBRT in oligometastatic PDAC cannot be precisely estimated.

Third, ctDNA-based patient selection—while mechanistically attractive and supported by emerging retrospective data—faces practical limitations in PDAC. Only 40–65% of patients with radiographically evident metastatic PDAC have detectable ctDNA using current clinical assays. The lead-time advantage of ctDNA over imaging may partially reflect assay sensitivity constraints rather than true biological advance warning. Prospective validation in PDAC-specific cohorts is essential before ctDNA-guided SBRT selection can be recommended outside clinical trials.

Fourth, the cost-effectiveness of SBRT—particularly MR-guided adaptive SBRT, which commands a substantial capital and operational premium over conventional linac-based delivery—has not been evaluated in oligometastatic PDAC. Health technology assessment data are urgently needed to inform resource allocation, particularly in publicly funded healthcare systems.

## 7 Limitations

**Figure 1:**
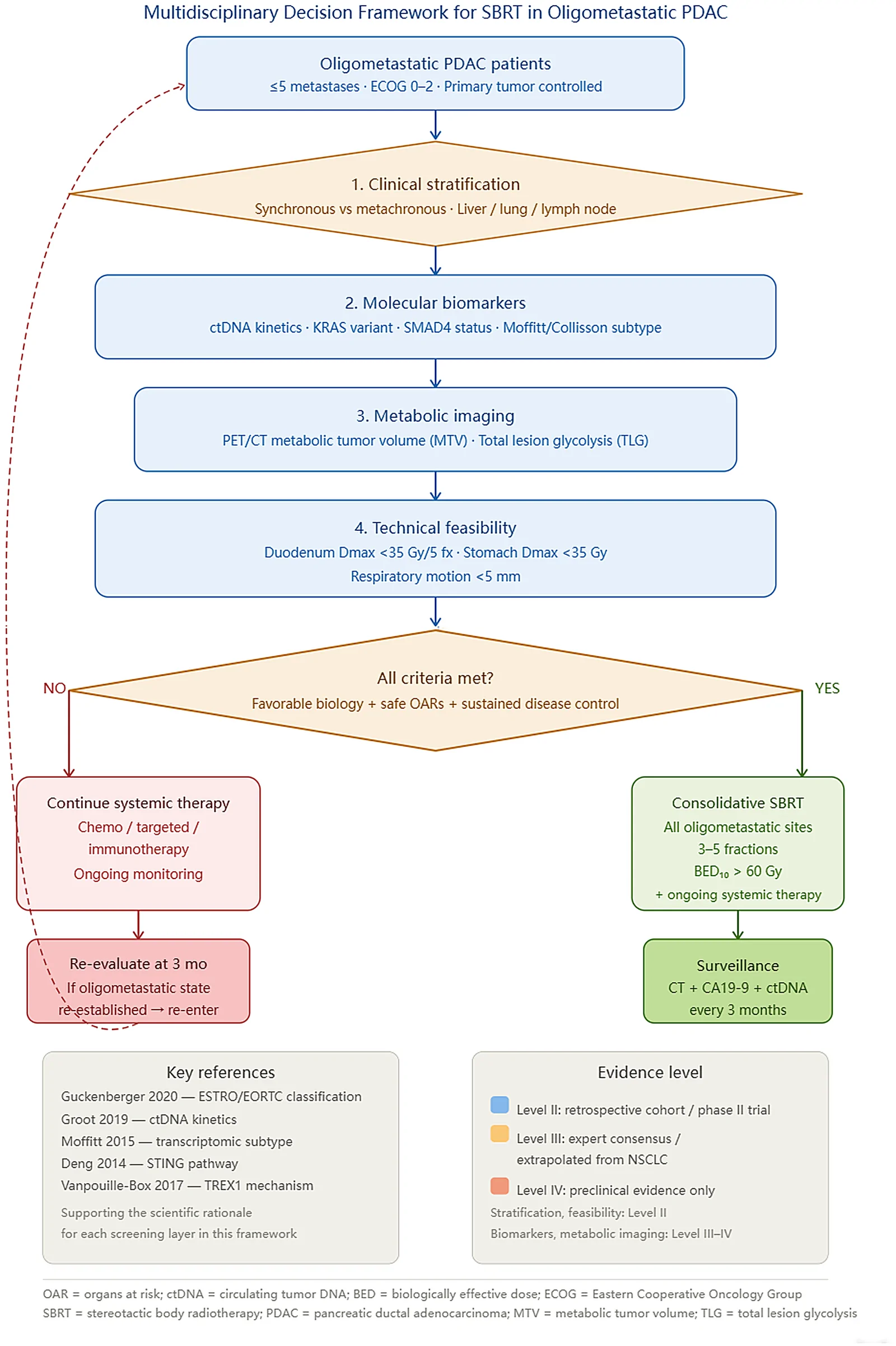
Proposed multidisciplinary decision framework for SBRT in oligometastatic PDAC. The algorithm integrates clinical stratification (synchronous vs metachronous, metastatic site), molecular biomarkers (ctDNA dynamics, KRAS subtype, transcriptomic classifier), metabolic imaging (PET/CT MTV/TLG), and technical feasibility (OAR geometry, motion amplitude) into a unified treatment recommendation. Patients who do not meet criteria for consolidative SBRT proceed with systemic therapy alone and are reassessed at 3-month intervals for possible conversion to an oligometastatic state amenable to local therapy.

This review has several limitations. First, this remains a narrative synthesis; formal meta-analysis was precluded by marked heterogeneity in study designs, populations, and outcome definitions. Second, the evidence base consists predominantly of single-institution retrospective series with inherent selection bias—patients selected for SBRT are systematically fitter than those receiving systemic therapy alone. Third, several recommendations concerning molecular biomarkers and immunotherapy combinations are extrapolated from preclinical or early-phase data and require PDAC-specific validation. Fourth, Asian-language literature was not comprehensively searched. Fifth, cost-effectiveness analyses are entirely absent. Finally, the proposed decision framework (**Figure 1**) remains hypothesis-generating and has not been prospectively validated.

## 8 Discussion

Radiotherapy for oligometastatic pancreatic ductal adenocarcinoma has moved decisively beyond its historical role in symptom palliation. The convergence of several developments—the validation of the oligometastatic paradigm across tumor types, the availability of effective systemic therapy regimens that can reduce and control metastatic burden, the technical maturity of SBRT delivery (particularly MR-guided adaptation and proton therapy), and the emerging understanding of how radiation modulates the tumor microenvironment—has created a rational framework for integrating radiotherapy as a potentially outcome-modifying component of multidisciplinary treatment.

Central to this paradigm is the recognition that not all patients with limited metastatic disease derive equivalent benefit from aggressive local therapy. Optimal outcomes depend on the intersection of three domains (1): favorable clinical and molecular biology (metachronous presentation, favorable subtype, ctDNA clearance during induction therapy) (2), technical feasibility of safe dose delivery to all target lesions (constrained by proximity to duodenum, stomach, and other serial OARs), and (3) effective integration with systemic therapy that addresses the underlying disease biology. Advances in each of these domains—from MR-guided adaptive SBRT and proton therapy to ctDNA-guided patient selection to the promising early signals of SBRT + immune checkpoint synergy—are broadening therapeutic window.

The limitations of the current evidence base must be acknowledged with candor. The field lacks a large-scale, phase III randomized controlled trial dedicated to pancreatic oligometastases. Retrospective data are subject to substantial selection bias. Quality of life and patient-reported outcomes—arguably the most patient-relevant endpoints in a disease with a 12–18 month median survival—are almost entirely absent from the literature. The identification of molecular and imaging biomarkers that reliably discriminate true oligometastatic biology from polymetastatic disease that merely appears oligometastatic on imaging remains an urgent unmet need.

Future progress requires a coordinated effort across radiation oncology, medical oncology, surgical oncology, and translational science. Dedicated clinical trials—not merely subgroup analyses of histology-agnostic studies—are essential. Systematic incorporation of PROs and QoL endpoints is non-negotiable. And prospective integration of liquid biopsies and molecular classifiers into patient selection algorithms will be critical to realizing the promise of precision radiation oncology in this challenging disease.

Radiotherapy in oligometastatic pancreatic cancer has evolved from palliation to disease-modifying multidisciplinary integration. The next decade will determine whether that evolution fulfills its potential.
